# Novel Dental Adhesive with Biofilm-Regulating and Remineralization Capabilities

**DOI:** 10.3390/ma10010026

**Published:** 2017-01-03

**Authors:** Yang Ge, Biao Ren, Xuedong Zhou, Hockin H. K. Xu, Suping Wang, Mingyun Li, Michael D. Weir, Mingye Feng, Lei Cheng

**Affiliations:** 1 State Key Laboratory of Oral Diseases, Sichuan University, Chengdu 610000, China; 2016324035015@stu.scu.edu.cn (Y.G.); renbiao@scu.edu.cn (B.R.); zhouxd@scu.edu.cn (X.Z.); 2015324035041@stu.scu.edu.cn (S.W.); limingyun@scu.edu.cn (M.L.); 2 Department of Operative Dentistry and Endodontics, West China Hospital of Stomatology, Sichuan University, Chengdu 610000, China; 3 Biomaterials & Tissue Engineering Division, Department of Endodontics, Periodontics and Prosthodontics, University of Maryland Dental School, Baltimore, MD 21201, USA; hxu@umaryland.edu (H.H.K.X.); MWeir@umaryland.edu (M.D.W.)

**Keywords:** dental caries, adhesive, quaternary ammonium monomers, poly (amidoamine) dendrimer, dimethylaminododecyl methacrylate

## Abstract

The mechanical properties and anti-caries effect of a novel anti-caries adhesive containing poly (amidoamine) dendrimer (PAMAM) and dimethylaminododecyl methacrylate (DMADDM) were investigated for the first time. Microtensile bond strength and surface charge density were measured for the novel anti-caries adhesives. *Streptococcus mutans*, *Streptococcus sanguinis*, and *Streptococcus gordonii* were chosen to form three-species biofilms. Lactic acid assay, MTT (3-(4,5-Dimethyl-thiazol-2-yl)-2,5-diphenyltetrazolium bromide) assay, exopolysaccharide staining and live/dead staining were performed to study anti-biofilm effect of the adhesive. The TaqMan realtime polymerase chain reaction was used to study the proportion change in three-species biofilms of different groups. The Scanning Electron Microscope (SEM) was used to observe the remineralization effect of PAMAM and DMADDM. The results showed that incorporating PAMAM and DMADDM into adhesive had no adverse effect on the dentin bond strength. The 1% PAMAM and 5% DMADDM adhesive group showed anti-biofilm properties and developed a healthier biofilm with a lower chance of inducing dental caries. Combination of 1% PAMAM and 5% DMADDM solution maintained remineralization capability on dentin, similar to that using 1% PAMAM alone. In conclusion, the adhesive containing PAMAM and DMADDM had strong antimicrobial properties and biological remineralization capabilities, and is promising for anti-caries clinical applications.

## 1. Introduction

Resin composites and adhesive systems are increasingly popular for tooth cavity restorations [1,2]. However, secondary caries at the restoration margins is considered as a main limitation of the longevity of the restorations [3]. Considering the increasing attention to the concept of minimally invasive cosmetic dentistry (MiCD), a new challenge to resin composites and adhesive systems is the possibility of a residual demineralization and a bacteria-containing layer in the cavity after preparation, which may cause a magnification of the problems associated with microleakage and lead to pulp inflammation [4]. Therefore, novel anti-caries adhesives with antibacterial and remineralization properties are needed.

Efforts were made to develop antibacterial adhesives by introducing antibacterial agents [5,6]. Quaternary ammonium monomers (QAMs) were investigated [7], including 12-methacryloyloxydodecylpyridinium bromide (MDPB) [1,8], quaternary ammonium dimethacrylate (QADM) [8,9] and dimethylaminododecyl methacrylate (DMADDM) [10,11].

Another method to obtain anti-caries adhesives was to incorporate remineralization agents into adhesive systems, including calcium phosphate compounds and biomimetic mineralization agents [12]. A previous study showed adhesive containing nanoparticles of amorphous calcium phosphate (NACP) could induce the formation of tertiary dentin in a rat cavity model [13]. However, a literature search revealed no reports on the incorporation of biomimetic mineralization agents such as poly (amidoamine) dendrimer (PAMAM) into adhesive. PAMAM was referred to as “artificial protein”, with a well-defined size, many reactive functional groups, and a controlled spatial structure [14,15]. PAMAM possessed the same templating and sequential functions as non-collagenous proteins (NCPs), which played a critical role in the biomineralization of dentin. In previous studies, the third generation PAMAM-NH_2_ (referred to as PAMAM in the present study), was shown to be a good template for inducing biomimetic remineralization of dentin [16].

The objectives of this study were to incorporate PAMAM and DMADDM into an adhesive and to investigate, for the first time, the biofilm-regulating and remineralization effects of the novel adhesive and dentin bonding properties. It was hypothesized that (1) incorporating PAMAM and DMADDM had no adverse effect on the dentin bonding properties; (2) adhesive containing PAMAM and DMADDM could inhibit the growth and the metabolic activity and regulate the controlled three-species biofilm; and (3) a combination of PAMAM and DMADDM solution could have a remineralization capability on dentin.

## 2. Results

### 2.1. Microtensile Bond Strength Test and Surface Charge Density

**Figure 1:**
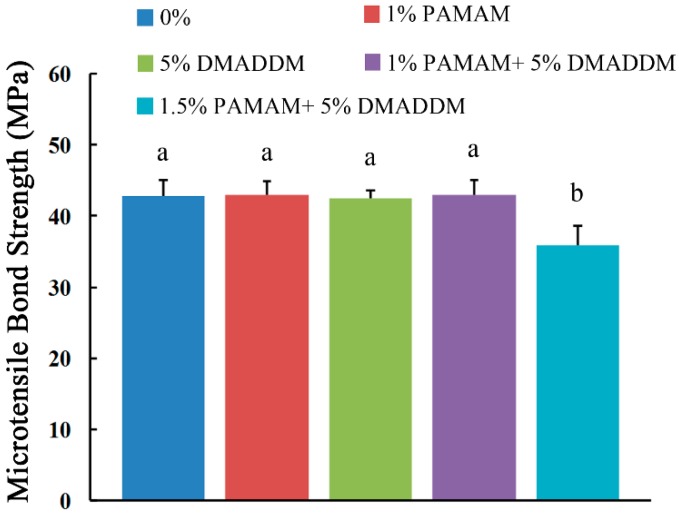
Microtensile bond strength of adhesive (mean ± sd; *n* = 6). Bars with the same letter indicate values that have no significant difference (*p* > 0.1) and those with the dissimilar letters indicate a significant difference (*p* < 0.05).

The microtensile bond strength results are plotted in Figure 1 (mean ± sd; *n* = 6). The adhesive containing 1% PAMAM, 5% DMADDM and 1% PAMAM + 5% DMADDM had bond strengths similar to that of the control group (*p* > 0.1). However, when adding 5% DMADDM and 1.5% PAMAM, the bond strength showed a significant decrease (*p* < 0.05), which is a crucial point for the selection of the tested concentrations.

**Figure 2:**
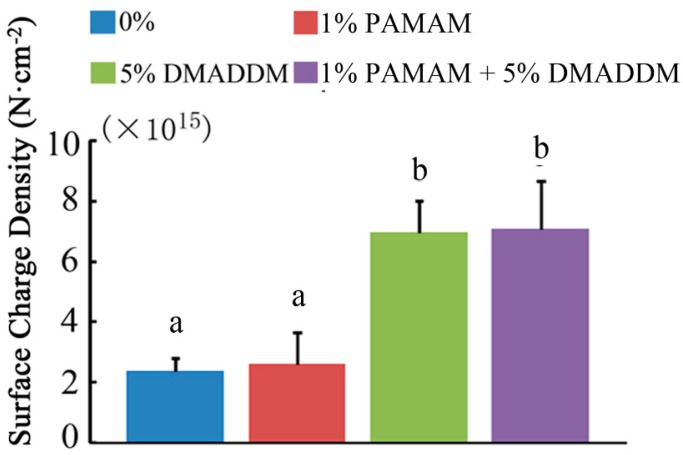
Surface charge density of the cured disks in four groups. Bars with the same letter indicate values that have no significant difference (*p* > 0.1) and those with the dissimilar letters indicate significant difference (*p* < 0.05).

Figure 2 shows the surface charge density of the cured adhesive disks. The “0%” showed the fluorescence bond to the vacant adhesive disks. Due to the amino group in PAMAM, there is a slight increase in the 1% PAMAM group (*p* > 0.1). When incorporated in DMADDM, the fluorescence increases significantly in the 5% DMADDM group (*p* < 0.05) and the 1% PAMAM + 5% DMADDM group (*p* < 0.05) due to the quaternary ammonium group. However, there is no significant difference between “5% DMADDM” and “1% PAMAM + 5% DMADDM” groups (*p* > 0.1).

### 2.2. Lactic Acid Measurement and MTT Assay

**Figure 3:**
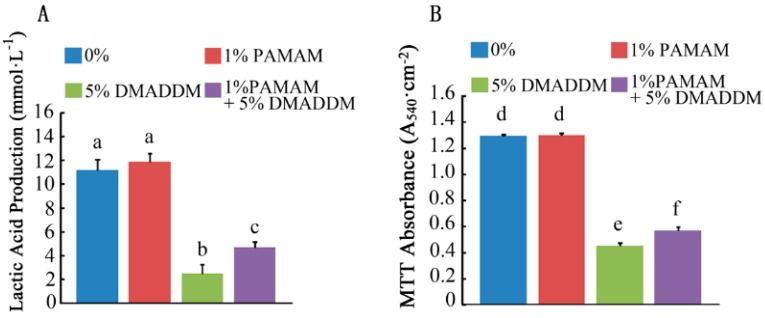
Lactic acid production and metabolic activity. (**A**) Lactic acid production by three-species biofilms adherent on the disks (mean ± sd; *n* = 6); (**B**) MTT assay of metabolic activity of biofilms adherent on the composites (mean ± sd; *n* = 6). Bars with the same letter indicate values that have no significant difference (*p* > 0.1) and those with the dissimilar letters indicate significant difference (*p* < 0.05).

The lactic acid production of biofilms is plotted in Figure 3A (mean ± sd; *n* = 6). Compared with the 0% group, the 1% PAMAM group showed no significant difference (*p* > 0.1). When DMADDM and PAMAM were incorporated, the 1% PAMAM + 5% DMADDM group showed a significant decrease in lactic acid production (*p* < 0.05), though it was slightly higher than the 5% DMADDM group alone (*p* < 0.05).

The MTT assay metabolic activity results of the three-species biofilms are plotted in Figure 3B (mean ± sd; *n* = 6). The 0% group and 1% PAMAM group had similar metabolic activity (*p* > 0.1). The 1% PAMAM + 5% DMADDM group significantly decreased the biofilm metabolic activity (*p* < 0.05). However, compared with the 5% DMADDM group, the 1% PAMAM + 5% DMADDM group had a higher metabolic activity (*p* < 0.05).

### 2.3. Live/Dead Bacteria Staining and Exopolysaccharide (EPS) Staining

**Figure 4:**
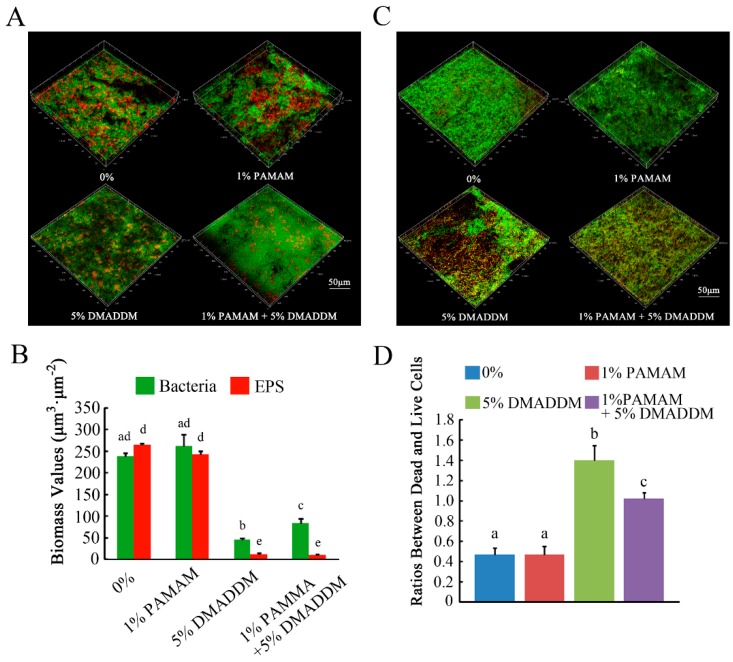
The micrographs of exopolysaccharide (EPS) staining and live/dead staining. (**A**) EPS staining of biofilms on the cured disks of the four groups. Bacteria were stained **green**, and EPS were stained **red**; (**B**) partly quantified analysis of bacteria and EPS production; (**C**) live/dead staining of biofilms on the cured disks of the four groups. Live cells were stained **green**, and dead cells were stained **red**; (**D**) Ratios between dead and live cells for four groups. Results were averaged from three randomly selected views of each group and are presented as mean ± standard deviation. Bars with the same letter indicate values that have no significant difference (*p* > 0.1) and those with the dissimilar letters indicate significant difference (*p* < 0.05).

Figure 4A shows the distribution of bacteria and EPS in the biofilms of the four groups. Bacteria were stained green, while EPS were stained red. In the 0% group, more bacteria and EPS were observed, similar to the 1% PAMAM group. In the 1% PAMAM + 5% DMADDM group, the bacteria and EPS were reduced significantly, but not less than those in the 5% DMADDM group. Figure 4B shows the results of relative fluorescence of bacteria and EPS production. Compared to the 0% and 1% PAMAM groups, bacteria and EPS production decreased in the 1% PAMAM + 5% DMADDM group. Representative Confocal Laser Scanning Microscope (CLSM) images of live/dead staining of biofilms are shown in Figure 4C. Live bacteria were stained green, while dead bacterial were stained red. Yellow color appeared because of the limitation of staining kits [17]. The ratios between dead and live bacteria computed with three randomly selected views of each group were plotted in Figure 4D.

### 2.4. Real-Time Polymerase Chain Reaction (RT-PCR)

**Figure 5:**
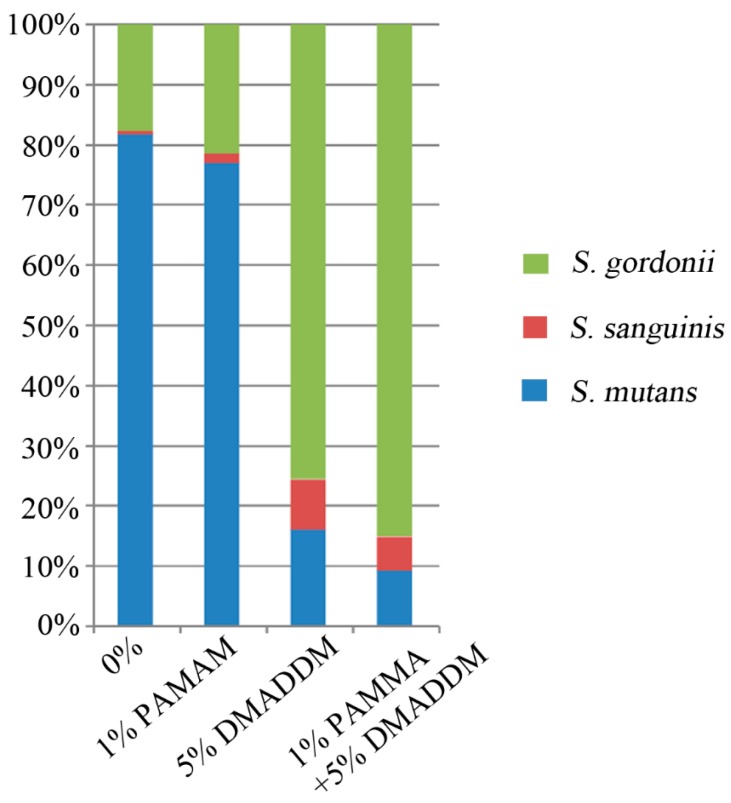
The ratios of *S. mutans*, *S. sanguinis*, and *S. gordonii* in three-species biofilms, conducted by TaqMan real-time polymerase chain reaction.

The RT-PCR results are plotted in Figure 5. In the 0% group, there was an increasing proportion of *Streptococcus mutans* in biofilms, with the percentage reaching 81.74%. The proportions of *Streptococcus sanguinis* and *Streptococcus gordonii* were only 0.52% and 17.74%, respectively. In the 1% PAMAM group, the proportion of *S. mutans* was also increasing to 77.09%, not higher than that in the 0% group, while the proportions of *S. sanguinis* and *S. gordonii* increasing to 1.48% and 21.43%. In the 5% DMADDM group, there was a significant decrease of the proportion of *S. mutans* to 16.11%, while the proportions of *S. sanguinis* and *S. gordonii* increased to 8.43% and 75.47%. Interestingly, in the 1% PAMAM + 5% DMADDM group, the proportion of *S. mutans* decreased to 9.34%, while the proportions of *S. sanguinis* and *S. gordonii* significantly increased to 5.57% and 85.10%, respectively.

### 2.5. Remineralization Effect Observed by Scanning Electron Microscope (SEM)

**Figure 6:**
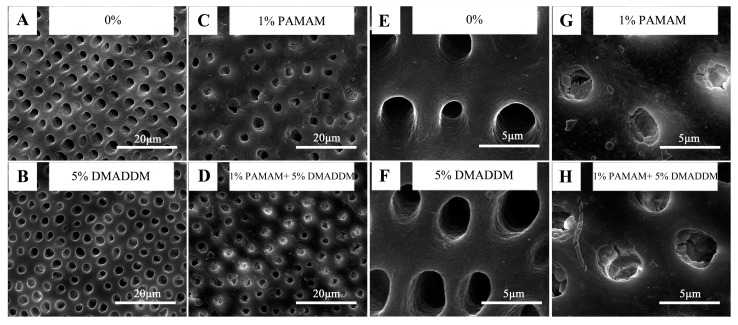
Scanning electron microscope (SEM) micrographs of remineralization effect: in (**A**,**B**), there were no minerals in dentin tubules. However, in (**C**,**D**), new minerals could be detected in dentin tubules after 14 days of immersion in artificial saliva solution. (**E**–**H**) are the higher magnification of (**A**–**D**), respectively, to show the tubules more obviously.

The remineralization effect of the dentin disks is shown in Figure 6. After 14 days of immersion in artificial saliva solution, there was nothing in dentin tubules in the 0% group, similar to the 5% DMADDM group. However, in both the 1% PAMAM and 1% PAMAM + 5% DMADDM group, the dentin tubules were filled with newly formed minerals, justifying that DMADDM has no side effect on the remineralization ability of PAMAM.

## 3. Discussion

The present study investigated the anti-caries effect of an adhesive containing PAMAM and DMADDM on the controlled three-species biofilms for the first time. The results indicated that the adhesive containing both PAMAM and DMADDM significantly inhibited lactic acid production and the growth of the three-species biofilms, the metabolic activity, and EPS synthesis. Moreover, the incorporation of PAMAM and DMADDM regulated the bacteria ratio of the three-species biofilms and help to develop a biofilm with lower cariogenic *S. mutans* proportion and reduced possibility of dental caries. Previous studies showed that it was favorable for adhesive to inhibit or kill residual bacteria in the prepared tooth cavity, as well as invading bacteria at the tooth-restoration interface through marginal leakage [18,19]. In the present study, the adhesive containing 5% DMADDM and 1% PAMAM + 5% DMADDM had strong antibacterial effect, which was consistent with previous studies [20,21].

PAMAM was incorporated into the adhesive to obtain biomimetic remineralization effect for the first time. Previous studies showed that some calcium phosphate compounds were only suitable for remineralizing the partially-demineralized dentin, which relied on the epitaxial deposition of calcium and phosphate ions over remnant crystallites [12,17]. In some clinical situations, dentin is completely demineralized and the collagen fibril is exposed. Traditional remineralization agents are ineffective as there are no remnant crystallites in the collagen fibrils. However, PAMAM was found to have the ability to remineralize not only the partially-demineralized dentin, but also collagen fibrils. This is because PAMAM is a biomimetic analog to NCPs and can induce biomimetic remineralization based on a particle-based crystallization concept [12,22]. PAMAM can perform its function when bonded to a surface and dentin collagen fibers. The SEM observation was performed to simply attest that DMADDM had no counteractive on the remineralization ability of PAMAM. Furthermore, in vivo experiments with adhesive containing PAMAM and DMADDM will be done in the future.

Regarding the anti-biofilm mechanism of DMADDM, there are two hypotheses. One is the contact-killing mechanism, which is the alteration of membrane permeability or surface electrostatic balance of bacteria, thus causing cytoplasmic leakage [23,24]. The other is that QAMs may serve as a trigger for programmed cell death in the surrounding bacteria [25,26]. In the present study, PAMAM showed no anti-biofilm effect, and adding PAMAM had some adverse effects on the anti-biofilm efficacy of DMADDM. However, the charge density measurement showed no difference between the DMADDM group and the PAMAM + DMADDM group. This implies that PAMAM might disturb some effects of DMADDM on the programmed cell death in the biofilms. Further study is needed to investigate why PAMAM slightly decreased the anti-biofilm efficacy of DMADDM in the PAMAM + DMADDM group.

*S. mutans*, *S. sanguinis* and *S. gordonii* are chosen for their presence in the oral cavity and the importance in the formation of dental plaque biofilm. *S. mutans* is associated with dental caries as a result of their competence of acid production and acid tolerance [26]. *S. mutans* binds to salivary agglutinin in the salivary pellicle that coats enamel and restorative surfaces. *S. sanguinis* is a common inhabitant in the human oral cavity, which was reported as an early colonizer during the formation of the dental plaque biofilm and is associated with low caries risk [27]. *S. sanguinis* is shown to antagonize *S. mutans* at the ecological level by competitively binding to salivary agglutinin and represents another major component of dental biofilm [28]. The three-species biofilm has been already used to investigate dental plaque biofilms in previous studies [11,28]. In a previous study, *S. mutans*, *S. gordonii*, and *S. sanguinis* were chosen to form a multi-species biofilm model, and the composition shift was shown to represent its developmental tendency to a healthy condition or to a caries risk [11]. The present study showed that the adhesive containing PAMAM and DMADDM regulated the biofilms to a healthy condition.

Previous studies showed there was no reduction in antibacterial and mechanical properties after water-aging for six months and one year [29,30] of adhesive containing DMADDM and NACP. It was attributed to the fact that DMADDM had covalent bonding to the resin polymer network, thus is immobilized and not lost or released from the adhesive resin over time. Furthermore, PAMAM has a nanoscopic size. However, the durability of the adhesive containing PAMAM and DMADDM still needs further investigation.

## 4. Materials and Methods

### 4.1. Synthesis of DMADDM and Specimen Preparation for Biofilm Experiments

DMADDM was synthesized via a modified Menschutkin reaction method, following previous studies [21]. PAMAM (CY Dendrimer Technology Company, Weihai, China) and DMADDM were incorporated into Clearfil SE Bond (Kuraray Dental, Tokyo, Japan) adhesive, at mass fractions according to the preliminary study of microtensile bond strength test. When DMADDM was added up to 5% (*wt*/*wt*), the microtensile bond strength would be compromised if PAMAM was added at a mass fraction of 1.5% (*wt*/*wt*) or higher. Therefore, four bonding systems were investigated in the study:

(1) Clearfil SE Bond control adhesive A (0%, control),
(2) A + 1% PAMAM (1% PAMAM),
(3) A + 5% DMADDM (5% DMADDM),
(4) A + 5% DMADDM + 1% PAMAM (1% PAMAM + 5% DMADDM).

The specimens for biofilm experiments were prepared according to the previous study [31]. Briefly speaking, the cover of a 48-well plate was used as the mold to fabricate the resin composites disks. Then, adhesive of different groups was used to cover the surfaces of the disks and light-cured for 10 s. After removing unpolymerized monomer by immersion in distilled water for 24 h, the specimens were sterilized in an ethylene oxide sterilizer (Anprolene AN 74i, Andersen, Haw River, NC, USA).

### 4.2. Microtensile Bond Strength Test

Microtensile bond strength test was performed according to a previous study [29]. After the ethical approval for the study was granted by the West China Hospital of Stomatology, Sichuan University, third molar teeth were collected and the informed consent was acquired from the donors. The midcoronal dentin was sectioned by a water-cooled low-speed saw (EMUC6/FC6, Leica Microsystems, Wetzlar, Germany) to obtain a flat dentin surface. The dentin surface was polished with 600-grit SiC paper. The segments were randomly divided into four groups (*n* = 6).

The bonding procedure was operated according to the specification uniformly. The flat dentin surface was disinfected with 75% ethanol and blown dry. The primer of SE Bond (Kuraray Dental, Tokyo, Japan) was applied with a brush-tipped applicator and rubbed for 20 s and dried with a strong stream of air. The adhesive of different groups was also applied to the dentin and dried for 5 s with a mild stream of air, followed by light-curing for 10 s. Then, AP-X resin composites (Kuraray Dental, Tokyo, Japan) were used to prepare the resin blocks on the dentin surface. The blocks were immersed in distilled water at 37 °C for 24 h. Then, the blocks were vertically sectioned into 1.0 mm × 1.0 mm × 12 mm composite-dentin beams. Each beam was stretched to tensile failure under computer-controlled Universal Testing Machine (MTS, Eden Prairie, MN, USA) at a cross-head speed of 1 mm/min immediately. The cross-sectional area at the site of failure was measured to calculate the microtensile bond strength values (MPa).

### 4.3. Surface Charge Density Test

The diameters and heights of three sterile disks for each group were measured, and the charge density of quaternary ammonium groups present on the polymer surfaces was quantified using fluorescein dye [32]. Furthermore, 1 mL fluorescein sodium salt (10 mg·mL^−1^ in deionized water) was added into each well of a 24-well plate with resin composites disks, and the plate was left at room temperature in the dark for 10 min. After rinsing completely in deionized water, each disk was transferred to a new well and 1 mL of 0.1% (by mass) cetyltrimethylamonium chloride (CTMAC) in deionized water was added to each well. The plate was shaken for 20 min at 80 rpm at room temperature in the dark to dissolve the bound dye. The CTMAC solution was supplemented with 100 μL 100 mM phosphate, pH 8.0 (0.94 mg·mL^−1^ monosodium phosphate-monohydrate and 13.2 mg/mL disodium phosphate-anhydrous in deionized water). In addition, 200 μL of the final solution was added into a 96-well plate, and sample absorbance was read at the OD_501nm_. Beers Law and an extinction coefficient of 77 mM^−1^·cm^−1^ were used to compute the fluorescein concentration. The ratio of accessible quaternary ammonium groups to fluorescein molecules is 1:1. The surface charge density was calculated as the total molecules of charge per exposed surface area. The tests were duplicated three times.

### 4.4. Bacteria Incubation and Biofilm Formation

*S. mutans* UA159, *S. sanguinis* SK1 and *S. gordonii* DL1 obtained from the State Key Laboratory of Oral Diseases (Sichuan University, Chengdu, China) were routinely cultured overnight in brain-heart infusion broth (BHI, Difco, Sparks, MD, USA) at 37 °C anaerobically (90% N_2_, 5% CO_2_, 5% H_2_).

To form three-species biofilms, bacterial suspensions were mixed with a defined microbial population consisting of *S. mutans* (10^7^ colony-forming units (CFUs)·mL^−1^), *S. sanguinis* (10^7^ CFUs·mL^−1^) and *S. gordonii* (10^7^ CFUs·mL^−1^) to obtain an inoculum. Furthermore, 200 μL of the mixture and 1.8 mL BHI supplemented with 1% sucrose were added into the 24-well plate, and each disk was placed in one well. The culture medium was refreshed every 24 h. The biofilms on the disks were cultured for 48 h at 37 °C anaerobically and used for further analysis.

### 4.5. Lactic Acid Measurement and MTT Assay

Followed by rinsing in phosphate buffered saline (PBS) to eliminate loose bacteria, six disks for each group with 48 h biofilm were put into a new 24-well plate with 1.5 mL buffered peptone water (BPW) and 0.2% sucrose in each well. The plate was cultured at 37 °C anaerobically for 3 h. After 3 h, the BPW solutions were left for lactate analysis, and the disks were transferred into a new 24-well plate for MTT assay. Lactate concentrations in the BPW solutions were measured using an enzymatic method [10]. A microplate reader (SpectraMax M5 (Molecular Devices, Sunnyvale, CA, USA) was used to monitor the absorbance at the OD_340__nm_.

The MTT (3-(4,5-Dimethyl-thiazol-2-yl)-2,5-diphenyltetrazolium bromide) assay was conducted according to a previous study [10]. Furthermore, 1 mL MTT dye (0.5 mg·mL^−1^ MTT in PBS) was incorporated into each well of the 24-well plate with the 48 h biofilms on the disks. The plate was cultured at 37 °C anaerobically for an hour. Then, the disks were transferred to a new 24-well plate with 1 mL dimethyl sulfoxide (DMSO) to dissolve the formazan crystals by shaking horizontally at 80 rpm for 20 min at room temperature in the dark. After being pipetted, 200 μL of the DMSO solution from each well was added into a 96-well plate, and the absorbance at the OD_540__nm_ was measured via the microplate reader. Both tests were repeated three times.

### 4.6. EPS Staining and Live/Dead Bacteria Staining

Three disks with 48 h biofilm were dyed with SYTO 9 and Alexa Fluor 647-dextran conjugate for EPS staining. The polysaccharides were labeled with Alexa Fluor 647-dextran conjugate (Molecular Probes, Invitrogen Corp., Carlsbad, CA, USA) to perform red fluorescence, while the bacterial cells were labeled by SYTO 9 green fluorescent nucleic acid stains (Molecular Probes, Invitrogen Corp., Carlsbad, CA, USA).

For live/dead staining, the disks with 48 h biofilms were stained using the BacLight live/dead bacterial viability kit (Molecular Probes, Eugene, OR, USA). Live bacteria cells were stained to produce green fluorescence with SYTO 9, while cells with compromised membranes were stained to red with propidium iodide.

Three disks were examined using confocal laser scanning microscopy (Leica, Wetzlar, Germany). Three disks for each group were examined and the tests were repeated for three times. The ratios between dead and live bacterial cells and partly quantified analysis of EPS production and bacteria cells were performed with Image-Pro Plus 6.0 (Media Cybernetics, Bethesda, MD, USA) and Matrix Laboratory (Mathworks, MA, USA) by calculating the value of relative fluorescence.

### 4.7. SEM Observation

The remineralization effect of PAMAM and DMADDM was also observed by SEM. The midcoronal dentin was sectioned by a water-cooled low-speed saw (EMUC6/FC6, Leica Microsystems, Wetzlar, Germany) and sectioned into 4.0 mm × 4.0 mm × 1.0 mm dentin disks and polished with 1000-grit SiC paper. The disks were divided into four groups randomly (*n* = 6). A 0.5 M EDTA (Ethylene diamine tetraacetic acid disodium salt) solution (pH 7.4) was used to prepare dentin erosion for 5 min as a previous study showed [12]. Then, dentin disks were rinsed with deionized water (DI) water and ultrasonically cleaned for 10 min. The DI water, DI water with 1% (*wt*/*wt*) PAMAM, with 5% (*wt*/*wt*) DMADDM, and with combination of 1% (*wt*/*wt*) PAMAM and 5% (*wt*/*wt*) DMADDM were prepared, respectively. The disks were applied with the four solutions above and air-dried 3 times. Then, the disks were immersed into artificial saliva solution for 14 days. The artificial saliva solution was refreshed every 24 h [12]. After 14 days, the dentin disks were air-dried and sputter-coated with gold examining via SEM Quanta 200 (FEI, Hillsboro, OR, USA).

### 4.8. DNA Isolation and Real-Time Polymerase Chain Reaction

Total DNA of biofilms on three disks of each group was isolated and purified using a TIANamp Bacteria DNA kit (TIANGEN, Beijing, China) according to the manufacturer’s instructions. The bacteria were lysed using enzymatic lysis buffer (20 mM Tris-HCl, pH 8.0; 2 mM sodium EDTA and 1.2% Triton X-100) containing 30 mg·mL^−1^ of lysozyme at 37 °C for 1 h. The purity and concentration of DNA were detected by NanoDrop 2000 spectrophotometer (Thermo Scientific, Waltham, MA, USA). The extracts were stored at −20 °C before use. TaqMan real-time polymerase chain reaction (Life Technologies, Carlsbad, CA, USA) was used to quantify the absolute number of *S. mutans*, *S. gordonii*, and *S. sanguinis* according to the manufacturer (Takara, Dalian, China), as a previous study showed [11]. For each real-time PCR, 20 μL mixture containing 10 μL of TaqMan Universal PCR Premix Ex Taq, 1.5 μL of template, 250 nM (each) of forward and reverse primer, and 250 nM of TaqMan probe were placed in each well. The sequences of primers and probes were listed in Table S1. Real-time PCR were performed as follows: 95°C for 3 min, followed by 40 cycles of 95 °C for 10 s and 56 °C for 30 s. The standard curves (Figure S1) of these bacteria were plotted using threshold cycle values obtained by amplifying successive 10-fold dilutions of known concentrations of DNA, which stands for corresponding concentrations of bacteria from 10^9^ CFUs to 10^4^ CFUs. The numbers of 3 strains were calculated based on standard curves generated using respective standard strains.

### 4.9. Statistical Analysis

One-way analyses of variance (ANOVA) were performed to detect the significant effects of the variables. Turkey’s multiple comparison was used to compare data at a *p*-value of 0.05.

## 5. Conclusions

The new adhesive containing PAMAM and DMADDM inhibited three-species biofilms growth, lactic acid production, metabolic activity and EPS production with biofilm-regulating effect. Incorporating PAMAM and DMADDM had no adverse effects on dentin bond strength. A combination of PAMAM and DMADDM produced a remineralization effect on dentin. The novel adhesive containing PAMAM and DMADDM with dual anti-caries effects is promising for a wide range of dental applications.

## Supplementary Materials

The following are available online at www.mdpi.com/1996-1944/10/1/26/s1. Figure S1: The standard curve plotted by known concentration of DNA of three species bacteria; Table S1: The sequences of primers and probes used in the study.

## References

[1] Imazato S. (2003). Antibacterial properties of resin composites and dentin bonding systems. Dent. Mater..

[2] Mmond J.L. (2008). Degradation, fatigue, and failure of resin dental composite materials. J. Dent. Res..

[3] Selwitz R.H., Ismail A.I., Pitts N.B. (2007). Dental caries. Lancet.

[4] Bunek S.S. (2014). Using minimally invasive techniques for the management and detection of dental caries. J. Cosmet. Dent..

[5] Martinez-Gutierrez F., Boegli L., Agostinho A., Sánchez E.M., Bach H., Ruiz F., James G. (2013). Anti-biofilm activity of silver nanoparticles against different microorganisms. Biofouling.

[6] Behnke S., Camper A.K. (2012). Chlorine dioxide disinfection of single and dual species biofilms, detached biofilm and planktonic cells. Biofouling.

[7] Ge Y., Wang S., Zhou X., Wang H., Xu H.H.K., Cheng L. (2015). The use of quaternary ammonium to combat dental caries. Materials.

[8] Imazato S. (2009). Bio-active restorative materials with antibacterial effects: New dimension of innovation in restorative dentistry. Dent. Mater. J..

[9] Cheng L., Weir M.D., Zhang K., Wu E.J., Xu S.M., Zhou X., Xu H.H.K. (2012). Dental plaque microcosm biofilm behavior on calcium phosphate nanocomposite with quaternary ammonium. Dent. Mater..

[10] Wang S., Zhang K., Xu N., Xu H.H.K., Weir M.D., Ge Y., Wang S., Li M., Li Y. (2014). Antibacterial effect of dental adhesive containing dimethylaminododecyl methacrylate on the development of streptococcus mutans biofilm. Int. J. Mol. Sci..

[11] Zhang K., Wang S., Zhou X., Xu H.H.K., Weir M.D., Ge Y., Li M., Wang S., Li Y., Xu X. (2015). Effect of antibacterial dental adhesive on multispecies biofilms formation. J. Dent. Res..

[12] Liang K., Yuan G., Li J., Ying L., Xiao S., Zhou X., Li J. (2015). Biomimetic mineralization of collagen fibrils induced by amine-terminated pamam dendrimers—pamam dendrimers for remineralization. J. Biomater. Sci. Polym. Ed..

[13] Li F., Wang P., Weir M.D., Fouad A.F., Xu H.H.K. (2014). Evaluation of antibacterial and remineralizing nanocomposite and adhesive in rat tooth cavity model. Acta Biomater..

[14] Svenson S., Tomalia D.A. (2005). Dendrimers in biomedical applications—Reflections on the field. Adv. Drug Deliv. Rev..

[15] Monica P., William T.B., Chunlin Q. (2010). Dentin sialophosphoprotein in biomineralization. Connect. Tissue Res..

[16] Liang K., Yuan H., Li J., Yang J., Zhou X., He L., Cheng L., Gao Y., Xu X., Zhou X. (2015). Remineralization of demineralized dentin induced by amine-terminated pamam dendrimer. Macromol. Mater. Eng..

[17] Sear R.P. (2013). The non-classical nucleation of crystals: Microscopic mechanisms and applications to molecular crystals, ice and calcium carbonate. Int. Mater. Rev..

[18] Reinke S.M.G., Divardin S., Raggio D., Reis A., Loguercio A.D. (2012). Degradation of the resin-dentin bonds after simulated and inhibited cariogenic challenge in an in situ model. J. Biomed. Mater. Res. B Appl. Biomater..

[19] Imazato S., Ma S., Chen J.H., Xu H.H.K. (2014). Therapeutic polymers for dental adhesives: Loading resins with bio-active components. Dent. Mater..

[20] Wang S., Ge Y., Zhou X., Xu H.H.K., Weir M.D., Zhang K., Wang H., Matthias H., Stefan R., Li Q. (2016). Effect of dimethylaminododecyl methacrylate containing glass-ionomer cement on the *S. Mutans* biofilms. Int. J. Oral Sci..

[21] Cheng L., Weir M.D., Zhang K., Arola D.D., Zhou X., Xu H.H.K. (2013). Dental primer and adhesive containing a new antibacterial quaternary ammonium monomer dimethylaminododecyl methacrylate. J. Dent..

[22] Liang K., Gao Y., Li J., Liao Y., Xiao S., Lv H., He L., Cheng L., Zhou X., Li J. (2014). Effective dentinal tubule occlusion induced by polyhydroxy-terminated pamam dendrimer in vitro. Rsc Adv..

[23] Namba N., Yoshida Y., Nagaoka N. (2009). Antibacterial effect of bactericide immobilized in resin matrix. Dent. Mater. Off. Publ. Acad. Dent. Mater..

[24] Beyth N., Yudovin-Farber I., Ran B., Domb A.J., Weiss E.I. (2006). Antibacterial activity of dental composites containing quaternary ammonium polyethylenimine nanoparticles against streptococcus mutans. Biomaterials.

[25] Zhou H., Weir M.D., Antonucci J.M., Schumacher G.E., Zhou X.D., Xu H.H. (2014). Evaluation of three-dimensional biofilms on antibacterial bonding agents containing novel quaternary ammonium methacrylates. Int. J. Oral Sci..

[26] Beyth N., Yudovin-Farber I., Perez-Davidi M., Domb A.J., Weiss E.I. (2010). Polyethyleneimine nanoparticles incorporated into resin composite cause cell death and trigger biofilm stress in vivo. Proc. Natl. Acad. Sci. USA.

[27] Ge Y., Caufield P.W., Fisch G.S., Li Y. (2008). Streptococcus mutans and streptococcus sanguinis colonization correlated with caries experience in children. Caries Res..

[28] Gregson K.S., Han S., Gregory R.L. (2012). The impact of three strains of oral bacteria on the surface and mechanical properties of a dental resin material. Clin. Oral Investig..

[29] Zhang K., Cheng L., Wu E.J., Weir M.D., Bai Y., Xu H.H.K. (2013). Effect of water-ageing on dentine bond strength and anti-biofilm activity of bonding agent containing new monomer dimethylaminododecyl methacrylate. J. Dent..

[30] Cheng L., Zhang K., Zhou C.C., Weir M.D., Zhou X.D., Xu H.H.K. (2016). One-year water-ageing of calcium phosphate composite containing nano-silver and quaternary ammonium to inhibit biofilms. Int. J. Oral Sci..

[31] Li F., Chen J., Chai Z.G., Zhang L., Xiao Y., Fang M., Ma S. (2009). Effects of a dental adhesive incorporating antibacterial monomer on the growth, adherence and membrane integrity of streptococcus mutans. J. Dent..

[32] Tiller J.C., Liao C.J., Lewis K., Klibanov A.M. (2001). Designing surfaces that kill bacteria on contact. Proc. Natl. Acad. Sci. USA.

