# Supplementary material for: Novel Dental Adhesive with Biofilm-Regulating and Remineralization Capabilities
Source: Materials (Basel). 2017 Jan 3;10(1):26. doi: 10.3390/ma10010026 (PMC5344622; doi:10.3390/ma10010026)
Supplement: Supplementary file 1 [file materials-10-00026-s001.pdf]

Supplementary Materials: Novel Dental Adhesive with Biofilm-Regulating and Remineralization Capabilities

Yang Ge, Biao Ren, Xuedong Zhou, Hockin H.K. Xu, Suping Wang, Mingyun Li, Michael D. Weir, Mingye Feng and Lei Cheng

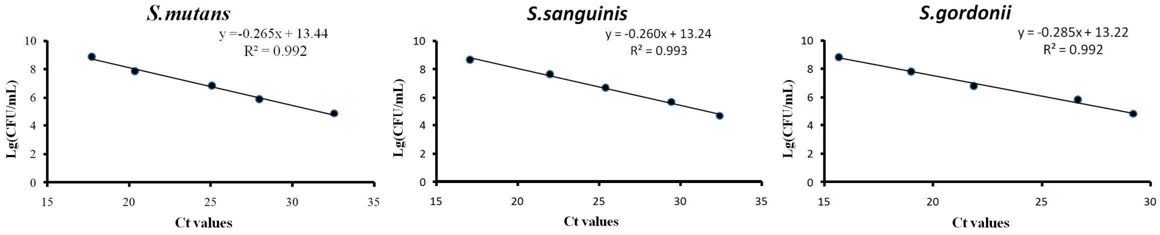

Figure S1. The standard curve plotted by known concentration of DNA of three species bacteria.

Table S1. The sequences of primers and probes.

Primers/Probes	Sequences	References
Primers		
S. mutans	F: 5′-GCCTACAGCTCAGAGATGCTATTCT-3′<br>R: 5′-GCCATACACCACTCATGAATTGA-3′	[1]
S. sanguinis	F: 5′-GAGCGGATGGCCAATTATATCT-3′<br>R: 5′-CCGGATGATGTCGGCAATA-3′	[2]
S. gordonii	F: 5′-GGTGTTGTTTGACCCGTTCAG-3′<br>R: 5′-AGTCCATCCCACGAGCACAG-3′	[3]
Probes		
S. mutans	5′-FAM-TGGAAATGACGGTCGCCGTTATGAA-TAMRA-3′	[1]
S. sanguinis	5′-FAM-AACCTTGACCCGCTCATTACCAGCTAGTATG-TAMRA-3′	[2]
S. gordonii	5′-FAM-TGTTCGGGCTCATGATA-Eclipse-3′	[3]

References

1 Suzuki, N.; Nakano, Y.; Yoshida, A.; Yamashita, Y.; Kiyoura, Y. Real-timeTaqMan PCR for quantifying oral bacteria during biofilm formation. *J. Clin. Microbiol.* **2004**, *42*, 3827–3830.

2 Zhang, K.; Wang, S.; Zhou, X.; Xu, H.H.K.; Wear, M.D.; Ge, Y.; Li, M.; Wang, S.; Li, Y.; Xu, X. Effect of antibacterial dental adhesive on multispecies biofilms formation. *J. Dent. Res.* **2015**, *94*, 622–629.

3 Yoshida, A.; Suzuki, N.; Nakano, Y.; Kawada, M.; Oho, T.; Koga, T. Development of a 5′ nuclease-based real-time PCR assay for quantitative detection of cariogenic dental pathogens Streptococcus mutans and Streptococcus sobrinus. *J. Clin. Microbiol.* **2003**, *41*, 4438–4441.
